# Semi-Supervised Methods to Predict Patient Survival from Gene Expression Data

**DOI:** 10.1371/journal.pbio.0020108

**Published:** 2004-04-13

**Authors:** Eric Bair, Robert Tibshirani

**Affiliations:** **1**Department of Statistics, Stanford UniversityPalo Alto, CaliforniaUnited States of America; **2**Department of Heath and Research Policy, Stanford UniversityPalo Alto, CaliforniaUnited States of America

## Abstract

An important goal of DNA microarray research is to develop tools to diagnose cancer more accurately based on the genetic profile of a tumor. There are several existing techniques in the literature for performing this type of diagnosis. Unfortunately, most of these techniques assume that different subtypes of cancer are already known to exist. Their utility is limited when such subtypes have not been previously identified. Although methods for identifying such subtypes exist, these methods do not work well for all datasets. It would be desirable to develop a procedure to find such subtypes that is applicable in a wide variety of circumstances. Even if no information is known about possible subtypes of a certain form of cancer, clinical information about the patients, such as their survival time, is often available. In this study, we develop some procedures that utilize both the gene expression data and the clinical data to identify subtypes of cancer and use this knowledge to diagnose future patients. These procedures were successfully applied to several publicly available datasets. We present diagnostic procedures that accurately predict the survival of future patients based on the gene expression profile and survival times of previous patients. This has the potential to be a powerful tool for diagnosing and treating cancer.

## Abstract 2

Procedures that utilize both gene expression data and clinical data to identify subtypes of cancer can provide more accurate prognoses

## Introduction

### Predicting Patient Survival

When a patient is diagnosed with cancer, various clinical parameters are used to assess the patient's risk profile. However, patients with a similar prognosis frequently respond very differently to the same treatment. This may occur because two apparently similar tumors are actually completely different diseases at the molecular level (Alizadeh et al. 2000; Sorlie et al. 2001; van de Vijver et al. 2002; van't Veer et al. 2002; Bullinger et al. 2004; Lapointe et al. 2004).

The main example discussed in this paper concerns diffuse large B-cell lymphoma (DLBCL). This is the most common type of lymphoma in adults, and it can be treated by chemotherapy in only approximately 40% of patients (NHLCP 1997; Vose 1998; Coiffier 2001). Several recent studies used DNA microarrays to study the gene expression profiles of patients with DLBCL. They found that it is possible to identify subgroups of patients with different survival rates based on gene expression data (Alizadeh et al. 2000; Rosenwald et al. 2002; Shipp et al. 2002).

If different subtypes of cancer are known to exist, there are a variety of existing techniques that can be used to identify which subtype is present in a given patient (Golub et al. 1999; Hastie et al. 2001a; Hedenfalk et al. 2001; Khan et al. 2001; Ramaswamy et al. 2001; Nguyen and Rocke 2002a, 2002b; Shipp et al. 2002; Tibshirani et al. 2002; van de Vijver et al. 2002; van't Veer et al. 2002; Nutt et al. 2003). However, most of these techniques are only applicable when the tumor subtypes are known in advance. The question of how to identify such subtypes, however, is still largely unanswered.

There are two main approaches in the literature to identify such subtypes. One approach uses unsupervised learning techniques, such as hierarchical clustering, to identify patient subgroups. This type of procedure is called “unsupervised” since it does not use any of the clinical information about the patient. The subgroups are identified using only the gene expression data. (In contrast, “supervised learning” would use the clinical data to build the model.) For an overview of unsupervised learning techniques, see Gordon (1999) or Hastie et al. (2001b).

Hierarchical clustering (Eisen et al. 1998) has successfully identified clinically relevant cancer subtypes in several different studies (Alizadeh et al. 2000; Bhattacharjee et al. 2001; Sorlie et al. 2001; Beer et al. 2002; Lapointe et al. 2004). However, one drawback to unsupervised learning procedures is that they may identify cancer subtypes that are unrelated to patient survival. Although several different subtypes of a given cancer may exist, if the prognosis for all patients is the same regardless of which subtype they have, then the utility of this information is limited. Since unsupervised learning procedures by definition do not use the clinical data to identify subtypes, there is no guarantee that the subtypes they identify will be correlated with the clinical outcome.

The second approach to identifying subtypes of cancer is based exclusively on the clinical data. For example, patients can be assigned to a “low-risk” or a “high-risk” subgroup based on whether they were still alive or whether their tumor had metastasized after a certain amount of time. This approach has also been used successfully to develop procedures to diagnose patients (Shipp et al. 2002; van de Vijver et al. 2002; van't Veer et al. 2002).

**Figure 1:**
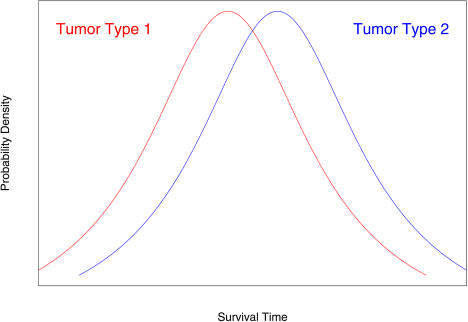
Two Patient Subgroups with Overlapping Survival Times

However, by dividing the patients into subgroups based on their survival times, the resulting subgroups may not be biologically meaningful. Suppose, for example, that there are two tumor cell types. Suppose further that patients with cell type 2 live slightly longer than patients with cell type 1 but that there is considerable overlap between the two groups (Figure 1). Assume also that the underlying cell types of each patient are unknown. If we were to assign patients to “low-risk” and “high-risk” groups based on their survival times, many patients would be assigned to the wrong group, and any future predictions based on this model would be suspect. We can obtain more accurate predictions by identifying these underlying subtypes and building a model that can determine which subtype is present in future patients.

### Proposed Semi-Supervised Methods

To overcome these difficulties, we propose a novel procedure that combines both the gene expression data and the clinical data to identify cancer subtypes. The crux of the idea is to use the clinical data to identify a list of genes that correlate with the clinical variable of interest and then apply unsupervised clustering techniques to this subset of the genes.

For instance, in many studies, the survival times of the patients are known even though no tumor subtypes have been identified (Alizadeh et al. 2000; Bhattacharjee et al. 2001; Sorlie et al. 2001; Beer et al. 2002; Rosenwald et al. 2002; Shipp et al. 2002; van de Vijver et al. 2002; van't Veer et al. 2002; Nutt et al. 2003; Bullinger et al. 2004). We can calculate the Cox score for each gene in the expression data—the Cox score measures the correlation between the gene's expression level and patient survival—and consider only the genes with a Cox score that exceeds a certain threshold.

Once such a list of significant genes is compiled, there are several methods we can use to identify clinical subgroups. We can apply clustering techniques to identify subgroups of patients with similar expression profiles. Once such subgroups are identified, we can apply existing supervised learning techniques to classify future patients into the appropriate subgroup. In this study, we will use the “nearest shrunken centroids” procedure of Tibshirani et al. (2002), which is implemented in the package PAM (Tibshirani et al. 2003). For a brief description of the procedure, see “Materials and Methods.”

Sometimes, however, a continuous predictor of survival is desired. We also describe a supervised version of principal components analysis that can be used to calculate a continuous risk score for a given patient and identify subtypes of cancer. The resulting predictor performs very well when applied to several published datasets.

These two methods will produce satisfactory results in most datasets. However, we will describe some variations of these methods that can sometimes improve their performance. When we cluster a dataset using only a subset of the genes, it is important that we choose the correct subset of genes. Choosing the genes with the largest Cox scores is generally a good strategy, but this procedure sometimes selects some spurious genes. We will show that one can use partial least squares (PLS) to compute a “corrected” Cox score. Selecting the genes with the largest “corrected” Cox scores can produce better clusters than selecting genes with largest raw Cox scores. Additionally, we will describe two other continuous predictors of survival that we will call β˜ and γ^. For some problems, they are better predictors than the continuous predictor based on supervised principal components (Figures S1–S3). These methods are described in Protocol S1.

### Related Methods in the Literature

Ben-Dor et al. (2001) and von Heydebreck et al. (2001) attempt to identify biologically meaningful tumor subtypes from gene expression data by clustering on a subset of the genes. The important distinction between these methods and our semi-supervised clustering method is that our method uses the available clinical data to choose the subset of the genes that is used to perform the clustering. The methods of von Heydebreck et al. (2001) and Ben-Dor et al. (2001) do not use this clinical information. We will show that utilizing the available clinical data can improve the quality of the clustering.

There are also related methods for predicting the survival of cancer patients using gene expression data. Nguyen and Rocke (2002a) use a form of PLS to predict survival. Li and Luan (2003) use support vector machines (SVMs). However, a drawback of these methods is the fact that they use a combination of all of the genes to predict survival. Since the vast majority of the genes in a given dataset are unrelated to survival, the result is that many of the inputs to the model are superfluous, which reduces the predictive accuracy of the model. We will show that our semi-supervised methods, which use only a subset of the genes, generally perform better than these methods.

Moreover, in many applications, we would like to identify which genes are the best predictors of survival. These genes could be analyzed in the laboratory to attempt to discover how they influence survival. They could also be used to develop a diagnostic test based on immunostaining or reverse transcriptase PCR. For these applications, it is important to have a predictor of survival that is based on a small subset of the genes. This is another important advantage of our methods over existing methods.

Beer et al. (2002) utilized an ad hoc method that fit a series of univariate Cox proportional hazards models and took a linear combination of the resulting coefficients. A brief description of their method is given in Protocol S1. This method is similar to our methods in that it selects a relevant subset of genes by choosing the genes with the largest Cox scores. However, this method is a purely supervised procedure. It does not apply any unsupervised methods (such as clustering or principal components analysis) to this subset of genes to identify additional patterns in the data. We will show that our semi-supervised procedures generally perform better than this method.

### Summary

Our goal is to identify subtypes of cancer that are both clinically relevant and biologically meaningful. Suppose that we have 𝓃 patients, and we measure the expression level of *p* genes for each patient. (Note that 𝓃 ≫ *p*.) We assume that there are several different types (classes) of cancer, each of which responds differently to treatment, and each of which is distinct at the molecular level. Therefore, given a set of 𝓃 patients with different classes of cancer, we wish to train a classifier that can diagnose which type of cancer a future patient has, given the expression levels of the patient's *p* genes. We will show that it is possible to identify such subgroups using the semi-supervised learning techniques described in the previous paragraph, and that identification of such subgroups can enable us to predict the clinical outcome of cancer more accurately.

## Results

### Fully Unsupervised Clustering

As noted in the Introduction, we needed to assign each patient to a subgroup before we could apply nearest shrunken centroids. First, we applied an unsupervised 2-means clustering procedure to the DLBCL data of Rosenwald et al. (2002). This dataset consisted of measurements on 7399 genes from 240 patients. Of these 240 patients, 160 were used for training the model and 80 were reserved for validating the model. A survival time was given for each patient, which ranged between 0 and 21.8 y.

We compared the survival times of the two subgroups using a log-rank test. The log-rank test statistic was 0.7, with a corresponding *p-*value of 0.416. Thus, conventional clustering techniques failed to identify subgroups that differed with respect to their survival times. Subgroups identified using hierarchical clustering also did not differ with respect to survival (data not shown).

### Using Clinical Data Alone to Generate Class Labels

**Figure 2:**
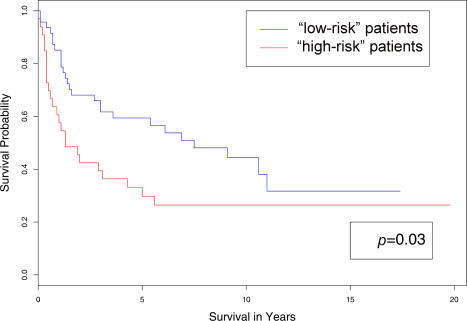
Comparison of the Survival Curves of the “Low-Risk” and “High-Risk” Groups These were obtained by applying nearest shrunken centroids to the DLBCL test data. Patients in the training data were assigned to either the “low-risk” or “high-risk” group depending on whether or not their survival time was greater than the median survival time of all the patients.

We assigned each patient in the training data to either a “low-risk” or “high-risk” subgroup based on their survival time (see “Materials and Methods” for details.) After applying nearest shrunken centroids with crossvalidation, we selected a model that used 249 genes. We then used this model to assign each patient in the independent test data to the “low-risk” or “high-risk” group. The plots of the two survival curves associated with the two classes generated by the model are shown in Figure 2. The *p*-value of the log-rank test was 0.03.

### Supervised Clustering

In order to identify tumor subclasses that were both biologically meaningful and clinically relevant, we applied a novel, supervised clustering procedure to the DLBCL data. We ranked all of the genes based on their univariate Cox proportional hazards scores, and performed clustering using only the “most significant” genes.

**Figure 3:**
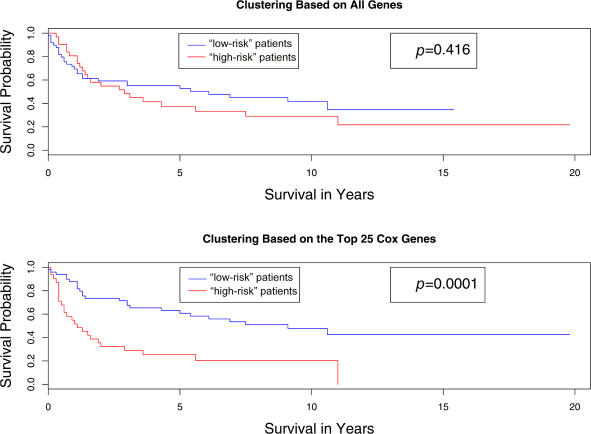
Comparison of the Survival Curves Resulting from Applying Two Different Clustering Methods to the DLBCL Data

Recall that when we performed 2-means clustering on the patients in the test data using all 7,399 genes and used a log-rank test to compare the survival times of the patients in the two resulting clusters, the result was not significant. To test our new clustering method, we calculated the Cox scores of all 7,399 genes based on the 160 training observations and ranked the genes from largest to smallest based on their absolute Cox scores. We then clustered the 80 test observations using only the 25 top-scoring genes. This time, the log-rank statistic comparing the survival times of the two clusters was highly significant ( *p* = 0.0001). A plot of the two resulting survival curves is shown in Figure 3. A plot of the survival curves that we obtained by applying 2-means clustering to all of the genes is also shown for comparison.

### Other Clustering Methods

Both Ben-Dor et al. (2001) and von Heydebreck et al. (2001) used a subset of the genes to try to cluster a microarray dataset in a biologically meaningful manner. They observed that clustering using a subset of the genes can produce better results than using all of the genes. However, these methods were still fully unsupervised since they used only the gene expression data to perform the clustering. They did not use the clinical data to identify subgroups.

Although these methods do a better job of identifying biologically meaningful clusters than clustering based on all of the genes, there is no guarantee that the clusters thus identified are associated with the clinical outcome of interest. Indeed, both Ben-Dor et al. (2001) and von Heydebreck et al. (2001) applied their procedures to a small DLBCL dataset of 40 patients (Alizadeh et al. 2000). The clusters they identified did not (with a few exceptions) differ significantly from one another with respect to survival.

**Figure 4:**
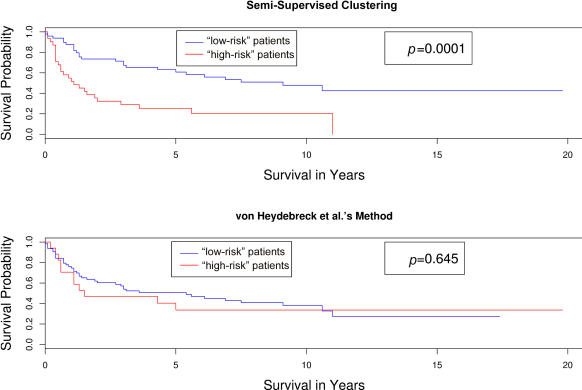
Comparison of the Survival Curves Resulting from Applying Two Different Clustering Methods to the DLBCL Data

We applied the clustering procedure of von Heydebreck et al. (2001) to the larger DLBCL dataset of Rosenwald et al. (2002). Figure 4 shows the survival curves of the two clusters generated using this method. The survival curves generated by clustering on the genes with the largest Cox scores are included for comparison. Note that the two subgroups identified using the clustering procedure of von Heydebreck et al. (2001) do not differ significantly with respect to survival.

### Survival Diagnosis

We showed that the cancer subgroups identified using this supervised clustering method can be used to predict survival in future patients. The idea is straightforward. First, we identified subgroups of patients using supervised clustering. Then we trained a nearest shrunken centroid classifier to predict the subgroup to which each patient belonged. Details are given in “Material and Methods.”

**Figure 5:**
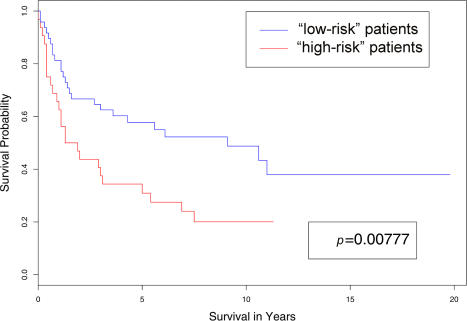
Survival Curves for Clusters Derived from the DLBCL Data

We tested this procedure on the DLBCL data. A clustering based on 343 genes produced the smallest crossvalidation error rate, so we used a classifier based on this clustering to assign each of the 80 test patients to one of the two subgroups. The survival curves of the two predicted subgroups are shown in Figure 5; the *p*-value of the log-rank test comparing the two survival curves is 0.008.

### Supervised Principal Components

We used a form of the principal components of the expression matrix to predict survival. Principal components analysis is an unsupervised learning technique that is used to reduce the dimensionality of a dataset by calculating a series of “principal components.” The hope is that the first few principal components will summarize a large percentage of the variability in the entire dataset. See Hastie et al. (2001b) for a description of principal components analysis.

Unfortunately, principal components analysis suffers from the same limitations as purely unsupervised clustering. If we perform principal components analysis using all of the genes in a dataset, there is no guarantee that the resulting principal components will be associated with survival. Thus, we propose a semi-supervised form of principal components analysis that we call “supervised principal components.” Rather than using all of the genes when we perform principal components analysis, we use only a subset of the genes that are correlated with survival.

Using the 160 training observations, we computed the Cox scores for each gene. We kept the 17 genes with Cox scores of 2.39 or greater. We calculated the principal components of the training data using only these 17 genes. Then we approximated the principal components of the test data using equation (11) (see “Materials and Methods” for details.)

**Figure 6:**
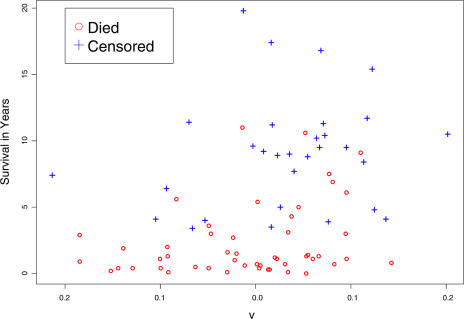
Plot of Survival Versus the Predictor υ^I for the DLBCL Data

Figure 6 shows that there does appear to be a correlation between the value of the first principal component, υ^I_1_, and patient survival. To confirm this observation, we fit a Cox proportional hazards model to a linear combination of υ^I_1_ and υ^I_2_, the estimated first and second principal components of the test data, respectively. (See “Materials and Methods” for a description of how this linear combination was obtained.) The resulting sum was a significant predictor of survival (*R* ^2^ = 0.113, likelihood ratio test statistic = 9.58, 1 d.f., *p* = 0.00197). This predictor is stronger than the discrete predictor shown in Figure 5 (*R* ^2^ = 0.08, likelihood ratio test statistic = 6.7, 1 d.f., *p* = 0.00966).

### A Breast Cancer Example

Thus far, all of our examples have been based on the DLBCL data of Rosenwald et al. (2002). We now apply our methodology to a set of breast cancer microarray data. In a recent study, van't Veer et al. (2002) built a model to predict the time to metastasis of breast cancer in patients based on microarray data from 78 patients. They showed that this model could be used to predict the times to metastasis of 20 independent test patients. Later, in a separate study, this same model was applied to a much larger set of 292 patients (van de Vijver et al. 2002).

Unfortunately, the expression levels of only 70 genes were available for the 292 patient dataset, making it difficult to test our methodology. However, we were able to apply our supervised principal components method. The expression levels of approximately 25,000 genes were available for the earlier study (consisting of 78 patients). After applying crossvalidation, we selected a model consisting of eight genes, five of which were included among the 70 genes in the larger dataset. Thus, we fit a supervised principal components model using these five genes and applied it to the dataset of 292 patients.

The results are shown in Table 1. (To compare the predictive power of the various models, we fit a Cox proportional hazards model to each predictor and computed the *R* ^2^ statistic for each model. *R* ^2^ measures the percentage of the variation in survival time that is explained by the model. Thus, when comparing models, one would prefer the model with the larger *R* ^2^ statistic.) We see that our supervised principal components method produced a stronger predictor of metastasis than the procedure described in van't Veer et al. (2002). Furthermore, our method used only five genes, whereas the predictor of van't Veer et al. (2002) used 70 genes. These results held even though we did not have the expression data for three genes that we would like to have included in our model.

**Table 1:**
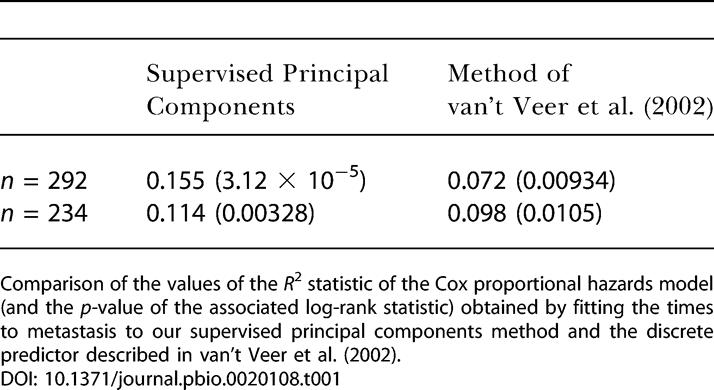
Supervised Principal Components Applied to Breast Cancer Data

Comparison of the values of the *R* ^2^ statistic of the Cox proportional hazards model (and the *p-*value of the associated log-rank statistic) obtained by fitting the times to metastasis to our supervised principal components method and the discrete predictor described in van't Veer et al. (2002)

(Of the 78 patients used to build the model in the original study, 61 were included in the larger dataset of 292 patients. Thus, the values of *R* ^2^ calculated using all 292 patients are inflated, since part of the dataset used to validate the model was also used to train the model. We include these results merely to demonstrate the greater predictive power of our methodology. Moreover, we repeated these calculations using only the 234 patients that were not included in the earlier study to ensure that our results were still valid.)

### Comparison With Related Methods in the Literature

We compared each of our proposed methods to several previously published methods for predicting survival based on microarray data. In particular, we examined three previously published procedures: a method based on SVMs (Li and Luan 2003), a method based on PLS (Nguyen and Rocke 2002a), and an ad hoc procedure that calculated a “risk index” for each patient by taking an appropriate linear combination of a subset of the genes (Beer et al. 2002). Finally, we considered a naive procedure that split the training data into two groups by finding the bipartition that minimized the *p*-value of the resulting log-rank statistic. A brief description of each of these procedures is given in Protocol S1; for a full description of these procedures, see the original papers.

We compared these methods on four different datasets (See Datasets S1-S13). First, we examined the DLBCL dataset (Rosenwald et al. 2002) that we used in the earlier examples. Recall that there were 7,399 genes, 160 training patients, and 80 test patients. Second, we considered a breast cancer dataset (van't Veer et al. 2002). There were 4,751 genes and 97 patients in this dataset. We partitioned this dataset into a training set of 44 patients and a test set of 53 patients. Third, we examined a lung cancer dataset (Beer et al. 2002). There were 7,129 genes and 86 patients, which we partitioned into a training set of 43 patients and a test set of 43 patients. Finally, we considered a dataset of acute myeloid leukemia patients (Bullinger et al. 2004). It consisted of 6,283 genes and 116 patients. This dataset was partitioned into a training set of 59 patients and a test set of 53 patients. The results are shown in Table 2.

**Table 2:**
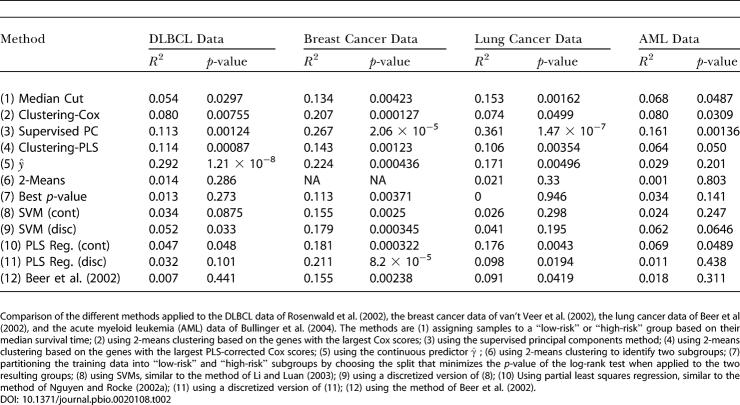
Comparison of the Different Methods on Four Datasets

Comparison of the different methods applied to the DLBCL data of Rosenwald et al. (2002), the breast cancer data of van't Veer et al. (2002), the lung cancer data of Beer et al (2002), and the acute myeloid leukemia (AML) data of Bullinger et al. (2004). The methods are (1) assigning samples to a “low-risk” or “high-risk” group based on their median survival time; (2) using 2-means clustering based on the genes with the largest Cox scores; (3) using the supervised principal components method; (4) using 2-means clustering based on the genes with the largest PLS-corrected Cox scores; (5) using the continuous predictor ; (6) using 2-means clustering to identify two subgroups; (7) partitioning the training data into “low-risk” and “high-risk” subgroups by choosing the split that minimizes the *p-*value of the log-rank test when applied to the two resulting groups; (8) using SVMs, similar to the method of Li and Luan (2003); (9) using a discretized version of (8); (10) Using partial least squares regression, similar to the method of Nguyen and Rocke (2002a); (11) using a discretized version of (11); (12) using the method of Beer et al. (2002)

### A Simulation Study

We compared each of the methods we proposed above on two simulated datasets. (See Data S1-S4.) The first simulated dataset *X* had 5,000 genes and 100 samples. All expression values were generated as standard normal random numbers with a few exceptions. Genes 1–50 in samples 1–50 had a mean of 1.0. We randomly selected 40% of the samples to have a mean of 2.0 in genes 51–100, 50% of the samples to have a mean of 1.0 in genes 101–200, and 70% of the samples to have a mean of 0.5 in genes 201–300.

We then generated survival times. The survival times of samples 1–50 were generated as normal random numbers with a mean of 10.0 and a standard deviation of 2.0, and the survival times of samples 51–100 were generated as normal random numbers with a mean of 8.0 and a standard deviation of 3.0. For each sample, a censoring time was generated as a normal random number with a mean of 10.0 and a standard deviation of 3.0. If the censoring time turned out to be less than the survival time, the observation was considered to be censored. Finally, we generated another 5000 × 100 matrix of test data X˜I, which was generated the same way *X* was generated. Survival times for X˜I were also generated in an identical manner.

We defined samples 1–50 as belonging to “tumor type 1” and samples 51–100 as belonging to “tumor type 2.” Thus, a successful subgroup discovery procedure should assign samples 1–50 to one subgroup, and samples 51–100 to the other subgroup.

We applied the methods discussed above to identify these subgroups (and predict survival) for the simulated dataset. This simulation was repeated ten times. The results are shown in Table 3. The first column of the table shows how many samples were misclassified when the dataset was originally divided into two subgroups. The second column shows the number of crossvalidation errors that occurred when the nearest shrunken centroids model was applied to these putative class labels. The third column shows the number of incorrectly labeled samples when the optimal nearest shrunken centroids model was used to assign labels to the samples in the test data X˜I. The final column is the value of *R* ^2^ obtained by fitting a Cox proportional hazards model to the predicted class labels for the test data (or by fitting a Cox model to γ^ in the case of methods 4 and 6).

**Table 3:**
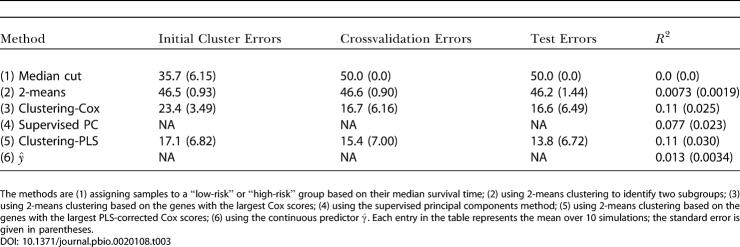
Comparison of the Different Methods on Our Simulated Data

The methods are (1) assigning samples to a “low-risk” or “high-risk” group based on their median survival time; (2) using 2-means clustering to identify two subgroups; (3) using 2-means clustering based on the genes with the largest Cox scores; (4) using the supervised principal components method; (5) using 2-means clustering based on the genes with the largest PLS-corrected Cox scores; (6) using the continuous predictor . Each entry in the table represents the mean over 10 simulations; the standard error is given in parentheses

In the first simulation, we found that the fully supervised and the fully unsupervised methods produced much worse results than the semi-supervised methods. (For each iteration of the “median cut” method, the crossvalidation error was minimized when all of the observations were assigned to the same class. Hence, each such model had no predictive power, and the value of *R* ^2^ was zero for each iteration. If we had chosen a smaller value of the tuning parameter Δ, the procedure would have performed better, although not significantly better.) The continuous predictor based on supervised principal components performed nearly as well as the methods based on semi-supervised clustering.

Next, we performed a second simulation. The second simulated dataset *X* had 1000 genes and 100 samples. All expression values were generated as Gaussian random variables with a mean of zero and a variance of 1.5, although again there were a few exceptions. Genes 1–50 had a mean of 0.5 in samples 1–20, a mean of 0.6 in samples 21–40, a mean of 0.7 in samples 41–60, a mean of 0.8 in samples 61–80, and a mean of 0.9 in samples 81–100. And again, we randomly selected 40% of the samples to have a mean of 2.0 in genes 51–100, 50% of the samples to have a mean of 1.0 in genes 101–200, and 70% of the samples to have a mean of 0.5 in genes 201–300. To generate the survival time of each “patient,” we took the sum of the expression levels of the first 50 genes and added a Gaussian noise term with variance 0.01. There was no censoring mechanism for the second simulation. We also generated another 1000 × 100 matrix of test data using an analogous procedure.

Under this model, there are actually five “tumor subgroups.” However, we still used 2-means clustering on this simulated dataset in order to evaluate the performance of our methods when the number of clusters is chosen incorrectly. Thus, in this simulation, it does not make sense to talk about the number of “misclassification errors;” we can only compare the methods on the basis of their predictive ability.

We applied the six different methods to this new simulated dataset and repeated this simulation ten times; the results are shown in Table 4. The supervised principal component method is the clear winner in the second simulation study. The semi-supervised methods performed poorly because there were a large number of subgroups and there was a considerable overlap between subgroups. This example demonstrates that the supervised principal component method performs well regardless of the number of tumor subclasses and that it seems to perform especially well when survival is an additive function of the expression level of certain genes.

**Table 4:**
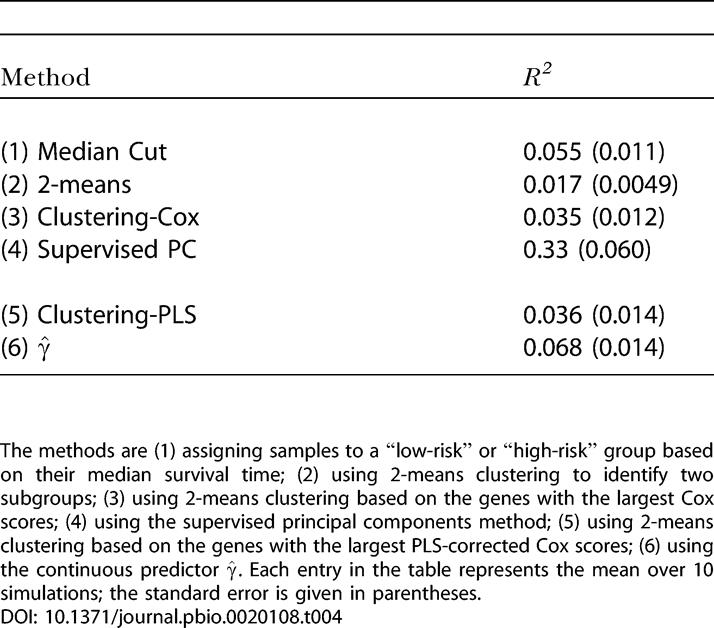
Comparison of the Different Methods on Our Simulated Data

The methods are (1) assigning samples to a “low-risk” or “high-risk” group based on their median survival time; (2) using 2-means clustering to identify two subgroups; (3) using 2-means clustering based on the genes with the largest Cox scores; (4) using the supervised principal components method; (5) using 2-means clustering based on the genes with the largest PLS-corrected Cox scores; (6) using the continuous predictor . Each entry in the table represents the mean over 10 simulations; the standard error is given in parentheses

## Discussion

One important goal of microarray research is to develop more powerful diagnostic tools for cancer and other diseases. Consider a hypothetical cancer that has two subtypes. One subtype is known to spread much more rapidly than the other subtype, and hence must be treated much more aggressively. We would like to be able to diagnose which type of cancer patients have and give them the appropriate treatment.

If it is known that two such subtypes of a certain cancer exist, and if we have a training set where it is known which patients have which subtype, then we can use nearest shrunken centroids or other classification methods to build a model to diagnose this cancer in future patients. However, in many cases, we do not know how many subtypes are present, nor do we know which patients belong to which subgroup. Thus, it is important to develop methods to identify such subgroups.

Unsupervised methods, such as hierarchical clustering, are popular techniques for identifying such subgroups. However, there is no guarantee that subgroups discovered using unsupervised methods will have clinical significance.

An alternative is to generate class labels using clinical data. The simplicity of the approach of dividing the patients into two subclasses based on their survival time is attractive, and there is evidence that this procedure can successfully predict survival. Indeed, this procedure produced a significant predictor of survival in four different datasets, suggesting that this approach has some utility. However, as noted in the Introduction, subgroups identified in this manner may not be biologically meaningful. When we applied this model to the DLBCL data described earlier, the misclassification error rate for the shrunken centroids model was very high (around 40%), so a diagnosis based on this procedure is likely to be inaccurate.

Supervised clustering methods can overcome these problems. We have seen that if we selected significant genes prior to clustering the data, we could identify clusters that were clinically relevant. We have also seen how knowledge of these clusters could be used to diagnose future patients.

This supervised clustering methodology is a useful prognostic tool. It is also easy to interpret. However, it has certain shortcomings as well. Recall our conceptual model shown in Figure 1. Patients with tumor type 2 live longer than patients with tumor type 1 on average, but there is still significant variability within each tumor type. Even if we can diagnose a patient with the correct tumor type 100% of the time, the prognosis of the patient may be inaccurate if the variability in survival time within each tumor type is large. Thus, it would be desirable to find a continuous predictor of survival that accounts for this within-group variability.

One possible such predictor is our supervised principal components procedure. This procedure used the principal components of a subset of the expression matrix *X* as a predictor of patient survival. The chosen subset contained the genes with the largest Cox scores. This method could also be used to detect cancer subtypes, since the principal components will presumably capture the variation that exists between subtypes. It is also capable of identifying variation within these subtypes, which, as discussed above, cannot be identified using supervised clustering. We showed that this procedure could produce a stronger predictor of survival than the discrete predictor based on supervised clustering.

We compared our methods to several previously published methods for predicting survival based on microarray data. In general, our methods performed significantly better than these existing methods. In particular, our supervised principal components method gave the best results on three of the four datasets. (It performed slightly worse than our γ^ method on the DLBCL data, but it still outperformed almost all of the other methods.) Furthermore, each of our proposed methods was a significant predictor of survival (at *p* = 0.05) for all four datasets, which was not true for any of the other methods. Finally, if we consider only discrete predictors of survival, our semi-supervised clustering methods performed better than the other models on at least three of the four datasets.

Another important advantage of our methods is that they select a subset of the genes to use as predictors. The methods of Nguyen and Rocke (2002a) and Li and Luan (2003), by contrast, require the use of all (or a large number) of the genes. If we can identify a small subset of genes that predict the survival of cancer patients, it may be possible to develop a diagnostic test using immunostaining or reverse transcriptase PCR. However, such a test would not be feasible if hundreds or thousands of genes were necessary to make the diagnosis.

Throughout this study, we have used survival data to help us identify genes of interest. However, other clinical variables could also be used, such as the stage of the tumor, or whether or not it has metastasized. Rather than ranking genes based on their Cox scores, one would use a different metric to measure the association between a given gene and the clinical variable of interest. For example, suppose we wished to identify a subgroup of cancer that was associated with a high risk of metastasis. For each gene, we could compute a t-statistic comparing the expression levels in the patients whose cancer metastasized to those in the patients with no metastasis. Tusher et al. (2001) described methods for generating such “significant gene lists” for a variety of possible clinical variables. Many of these methods are implemented in the significance analysis of microarrays software package (Chu et al. 2002).

Information about the risk of metastasis (and death) for a given patient is essential to treat cancer successfully. If the risk of metastasis is high, the cancer must be treated aggressively; if the risk is low, milder forms of treatment can be used. Using DNA microarrays, researchers have successfully identified subtypes of cancer that can be used to assess a patient's risk profile. Our results show that semi-supervised learning methods can identify these subtypes of cancer and predict patient survival better than existing methods. Thus, we believe they can be a powerful tool for diagnosing and treating cancer and other genetic diseases.

## Materials and Methods

### 

#### Overview of nearest shrunken centroids

The nearest shrunken centroids procedure calculates the mean expression of each gene within each class. Then it shrinks these centroids toward the overall mean for that gene by a fixed quantity, Δ. Diagonal linear discriminant analysis (LDA) is then applied to the genes that survive the thresholding. Details are given in Tibshirani et al. (2002). It has successfully classified tumors based on gene expression data in previous studies. In one experiment, there were a total of 88 patients, each of which had one of four different types of small round blue cell tumors . Nearest shrunken centroids classified 63 training samples and 25 test samples without a single misclassification error (Tibshirani et al. 2002).

#### Generation of “median cut” class labels**

We created two classes by cutting the survival times at the median survival time (2.8 y). Any patient who lived longer than 2.8 y was considered to be a “low-risk” patient, and any patient that lived less than 2.8 y was considered to be a “high-risk” patient. In this manner, we assigned a class label to each observation in the training data.

Unfortunately, many of the patients' survival times were censored, meaning that the individual left the study before the study was completed. When this occurs, we do not know how long the patient survived; we only know how long the patient remained in the study prior to being lost to follow-up.

If an observation is censored, we may not know to which class it belongs. For example, suppose that the median survival time is 2.8 y, but that a patient left the study after 1.7 y. If the patient died in the interval between 1.7 y and 2.8 y, then the patient should be assigned to the “high-risk” group. Otherwise, the patient should be assigned to the “low-risk” group. However, there is no way to determine which possibility is correct.

Based on the Kaplan-Meier survival curve for all the patients, we can estimate the probability that a censored case survives a specified length of time (Cox and Oakes 1984; Therneau and Grambsch 2000). For example, suppose that the median survival time is 50 months and a patient left the study after 20 months. Let *T* denote the survival time of this patient. Then, using the Kaplan-Meier curve, we can estimate *p*(*T*>50) and *p*(*T*>20). Then we can estimate *p*(*T*>50|*T*>20) as follows:



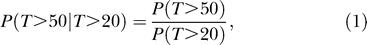



and, of course,



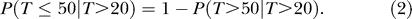



In this manner, we can estimate the probability that each censored observation belongs to the “low-risk” and “high-risk” classes, respectively.

However, it is still unclear how we would train our classifier based on this information. Nearest shrunken centroids is a modified version of LDA. It is described in detail in Hastie et al. (2001b). Like most classification techniques, LDA assumes that the class labels of the training observations are known with complete certainty. The version of LDA described in Hastie et al. (2001b) and most other books cannot handle probabilistic class labels, where there is a certain probability that a training observation belongs to one class, and a certain probability that it belongs to a different class. We will now describe a simple modification of LDA that can be trained based on this type of data. It is similar to a technique described in McLachlan (1992) for training an LDA classifier when some of the training observations are missing.

Let {**x**_*i*_}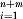 denote the set of input variables, and let {**y**_*i*_}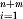 represent the corresponding response variables. Also, let *g* represent the number of discrete classes to which the *y*_*i*_s may belong. (If we are dividing the training data into “low-risk” and “high-risk” patients, then *g* = 2.) When we perform LDA when all of the *y*_*i*_s are known, the problem is to fit the mixture model



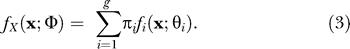



(Generally, each 𝒻_*i*_ is a Gaussian density function, and the θ_*i*_s correspond to the mean of the observations in each class. The π_*i*_s correspond to “prior” probabilities that an observation belongs to class 𝒾.) In this case, we must fit this model on the basis of the classified (uncensored) training data, which we denote by **t**, and the unclassified (censored) feature vectors **x**_𝒿_ ( 𝒿 = 𝓃+1, …,𝓃+𝓂), which we denote by **t**_𝓊_. (Also, note that Φ = (π′,θ′)′ denotes the vector of all unknown parameters.)

We define the latent variables 𝓏_*ij*_ to be equal to one if the 𝒿th observation belongs to the 𝒾th class, and zero otherwise. Then the complete-data log likelihood is



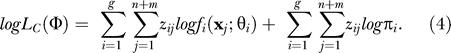



The EM algorithm is applied to this model by treating **z**_*j*_(𝒿 = 𝓃+1,…, 𝓃+𝓂) as missing data. It turns out to be very simple in the case of LDA. The E-step is effected here simply by replacing each unobserved indicator variable 𝓏_*ij*_ by its expectation conditional on **x**_*j*_. That is, 𝓏_*ij*_ is replaced by the estimate of the posterior probability that the 𝒿th entity with feature vector **x**_*j*_ belongs to *G*_*i*_(𝒾 = 1, …,*G*, 𝒿 = *n* + 1, …,*n*+*m*) (McLachlan 1992). We take the initial estimates of 𝓏_*ij*_ to be the earlier estimate that the 𝒾th censored observation belongs to class 𝒿 based on the Kaplan-Meier curve.

The estimates of π_*i*_ and μ_*i*_ in the M-step are equally simple:



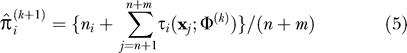



and



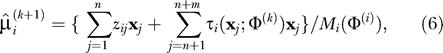



where



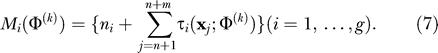



In these expressions, τ_*i*_(**x**;Φ) is the posterior probability that the 𝒿th entity with feature vector **x**_*j*_ belongs to *G*_*i*_, or, in other words,



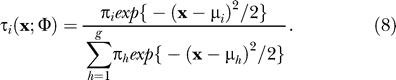



We continue these imputations until the algorithm converges. In practice, one imputation seems to be sufficient for most problems, since each imputation is computationally intensive, and additional imputations did not seem to change the results significantly.

#### Diagnosing patient survival via supervised clustering**

We calculated the Cox scores of each gene based on the 160 training observations, and obtained a list of the most significant genes. Then we performed 2-means clustering on these 160 observations using the genes with the largest absolute Cox scores and obtained two subgroups. We repeated this procedure multiple times with different numbers of genes. For each such clustering, we trained a nearest shrunken centroid classifier to assign future patients to one subgroup or the other and examined the crossvalidation error rate.

The problem of choosing the number of genes on which to perform the clustering is more complicated than it appears. The obvious way to choose the optimal number of genes on which to cluster is to simply minimize the crossvalidation error rate of the nearest shrunken centroids model based on the clustering. This works up to a certain point. It is possible that the clustering procedure will identify a cluster that is unrelated to survival. (Since we are clustering on the genes with the highest Cox scores, this is unlikely to occur. However, it is still possible, especially if the number of genes on which we are clustering is large.) Thus, we needed to build a safeguard against this possibility into our procedure. After performing clustering based on a given set of high-scoring genes, we performed a log-rank test to determine if the resulting clusters differed with respect to survival. If they did not, the clustering was discarded without further analysis. An outline of the procedure follows: (1) Choose a set *G* of possible values of Γ. (2) Let *p*_min_ = 1 and *e*_min_ = 1. (3) For each Γ in *G*, do the following: (4) Perform *k*-means clustering using only those genes with absolute Cox scores greater than Γ. (5) Perform a log-rank test to test the hypothesis that the *k* clusters have different survival rates. Call the *p*-value of this test *p*. (6) If *p*≥*p*_min_, then return to step 3. (7) Fit a nearest shrunken centroids model based on the clusters obtained in step 3. Calculate the minimum crossvalidation error rate across all values of the shrinkage parameter, and call it *e*. (8) If *e*<*e*_min_, then let Γ_best_ = Γ, and return to step 3. Otherwise return to step 3 without changing the value of Γ_best_. The optimal value of Γ is taken to be the value of Γ_best_ when this procedure terminates.

Several comments about this procedure are in order. First, note that we did not recalculate the Cox scores at each fold of the crossvalidation procedure. We calculated them only once, using all of the patients in the dataset. There are several reasons for doing this. Recalculating the Cox scores at each fold would be extremely expensive computationally. Moreover, we found that the Cox score of a given gene varied depending on the number of patients (and which patients) we included in the model. Thus, if a given value of Γ produced a low crossvalidation error rate, there was no guarantee that a model based on the full dataset using this value of Γ would produce good results, since the model based on the full dataset may use a different list of genes. Other studies have found that using the entire dataset to produce a “significant gene list” prior to performing crossvalidation can produce more accurate predictions (van't Veer et al. 2002).

Also, the set *G* was left unspecified in the procedure. The choice of which (and how many) possible values of Γ to include in *G* depends on the problem at hand, as well as the computational power available. As a default, we recommend trying 100 evenly spaced values of Γ between the 90th percentile of the Cox scores and the maximum of the Cox scores. However, the optimal Γ_best_ varies greatly from dataset to dataset, so we recommend trying several different forms of *G* if adequate computing power exists.

Furthermore, note that when we calculated the *p*-value of the log-rank test after performing the original clustering, we insisted not only that the *p*-value be significant, but also that it be lower than the best *p*-value obtained thus far. The reasons for this are twofold. First, experience suggests that if a given set of genes produces a good clustering on the training data (“good” defined as having a low *p*-value from a log-rank test), then it is likely to produce a good clustering on the test data. (We offer no theoretical or biological justification for this statement; it simply represents our experience. However, we have observed this result a sufficient number of times to convince us that it is not coincidental.) Moreover, this speeds up the algorithm substantially. Calculating the nearest shrunken centroids crossvalidation error rate for a given clustering is the slowest part of the procedure; the time required to perform the clustering and calculate the log-rank statistic is insignificant in comparison. Thus, by only considering clusterings which produce a log-rank statistic with a small *p*-value, we allow the set *G* to be much larger than would be feasible otherwise.

Finally, the number of clusters *k* was unspecified in the procedure. We have experimented with some algorithms to choose the value of *k* automatically, but without success. If possible, we recommend that the value of *k* be chosen based on prior biological knowledge. (Perhaps one could first perform hierarchical clustering, examine a dendogram of the data, and visually search for major subgroups.) If this is not possible, we recommend trying several different small values of *k* and choosing the one that gives the best results. (Our experience suggests that choosing *k* = 2 will give good results for almost all datasets.)

#### Supervised principal components**

As above, let *X* be the *p*×*n* matrix of expression values, for *p* genes and *n* patients. Let *x*_*ij*_ denote the expression level of the 𝒾th gene in the 𝒿th patient. Assume that each patient has one of two possible underlying tumor types. Without loss of generality, assume that patients 1, …,*m* have tumor type 1, and that patients *m* + 1,…,*n* have tumor type 2. Then assume that the genetic profiles of the two tumor types are distinct from one another, which is equivalent to assuming that the joint distribution of (*x*_1*j*_, …,*x*_*pj*_) is different for 1 ≤ 𝒿 ≤ 𝓂 than it is for 𝓂 + 1 ≤ 𝒿 ≤ 𝓃. Thus, if we choose constants {*a*_*i*_}^*p*^_*i*=1_, the distribution of ∑^*p*^_*j*=1_ *a_j_x_ij_* will be different for 1 ≤ 𝒿 ≤ 𝓂 than it is for 𝓂 + 1 ≤ 𝒿 ≤ 𝓃. (Obviously, this is not true for all values of {*a_i_*}^*p*^_*i*_=1. For example, if we let *a_i_* = 0 for all 𝒾, then this statement will not hold. However, it will generally be true unless we deliberately choose a pathological set {*a_i_*}^*p*^_*i*_=1.)

In particular, consider the singular value decomposition of *X*:



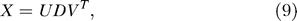



where *U* is a *p* × 𝓃 orthogonal matrix, *D* is an 𝓃 × 𝓃 diagonal matrix, and *V* is an 𝓃 × 𝓃 orthogonal matrix (Horn and Johnson 1985). Then the matrix *V* can be written as



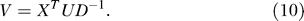



In other words, for a given column of *V*, each row of *V* is a linear combination of the expression values in the corresponding column of *X*. Thus, by the argument in the preceding paragraph, rows 1 through 𝓂 should have a different distribution than rows 𝓂 + 1 through 𝓃. Hence, we propose that the first few columns of *V* be used as continuous predictors of survival for each patient. Formally,



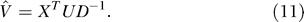



Moreover, suppose that we have an independent test set X˜I. Then let



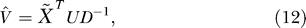



where *U* and *D* are the same as in equation (11) (i.e., derived from the singular value decomposition of the training data). In this case, the first few columns of V^I can be used to estimate survival for the patients in the independent test set.

The reason for choosing the first few columns of *V* is because the matrix *U* was chosen so that *X^T^u*_1_ has the largest sample variance amongst all normalized linear combinations of the rows of *X* (Hastie et al. 2001b). (Here, *u*_1_ represents the first column of *U*.) Hence, assuming that variation in gene expression accounts for variation in survival, we would expect that *X^T^*_*u*1_ captures a large percentage of the variation in survival. (Indeed, in some simple models, it can be proven that equation [11] is the best possible predictor of survival; see Protocol S1.)

In theory, we could calculate *V* using the entire dataset *X*, and the rows of *V* would have different distributions depending on the tumor type of the corresponding patient. In practice, however, many of the genes in *X* are unrelated to survival, and if we use the entire dataset *X* to compute *V*, the quality of the resulting predictor is poor. We can overcome this difficulty by using only the genes with the largest Cox scores. Formally, we construct a matrix *X*′ consisting of only those genes whose Cox scores are greater than some threshold Γ, and take the singular value decomposition of *X*′.

To choose the optimal value of Γ, we employ the following procedure: (1) Choose a set *G* of possible values of Γ. (2) For each Γ in *G*, split the training data into *k* random partitions (i.e., perform *k*-fold crossvalidations). For most problems (and for the rest of this discussion), we can let *k* = 10. (3) For each crossvalidation fold, take a singular value decomposition of *X*, leaving out one of the 10 partitions for validation purposes. Use only those genes with absolute Cox scores greater than Γ. (4) Calculate υ^ for the 10% of the data that was withheld, as described above. (5) Fit a Cox proportional hazards model to υ^, and calculate the chi-square statistic for the log-rank test associated with this model. Denote the chi-square statistic for the 𝒾th crossvalidation fold by 𝓌_*i*_. (6) Average the 𝓌_*i*_s over the 10 crossvalidation folds. Call this average 𝓌_Γ_. (7) If 𝓌_Γ_ is greater than the value of 𝓌_Γ∗_, then let Γ∗ = Γ and 𝓌_Γ∗_ = 𝓌_Γ_. (8) Return to step 2. The set *G* is left unspecified in the procedure. As a default, we recommend trying 30 evenly spaced values of Γ between the 90th percentile of the Cox scores and the maximum of the Cox scores, although this recommendation is somewhat arbitrary.

In some cases, we can improve the predictive power of our model by taking a linear combination of several columns of *V* (rather than simply taking the first column of *V*). Suppose we wish to find a predictor based on the first *k* columns of *V*. We can perform the following procedure: (1) Let *X* denote the training data. Take the singular value decomposition of *X* = *UDV^T^* as described above (after selecting an appropriate subset of the genes). (2) Fit a Cox proportional hazards model using the first *k* columns of *V* as predictors. (3) Calculate the matrix V^I for the test data using equation (12) above. (4) Take a linear combination of the first *k* columns of V^I using the Cox regression coefficients obtained in step 2. Use the resulting sum as a continuous predictor of survival.

#### Software and computational details**

All computations in this study were performed using the R statistical package, which is available on the Internet at http://cran.r-project.org/. R source code for the procedures described in this paper are available from the authors upon request (see also Data S1–Data S4). These methods will also be implemented in a future version of the PAM microarray analysis package (Tibshirani et al. 2003). (The “median cut” method has been implemented in version 1.20, which is now available.)

## Supporting Information

Data S1 Documentation of Our R Functions This file contains a brief description of the functions contained in the semi-super.R file.
(1 KB TXT).

Data S2 R Source Code This file contains R functions for implementing the procedures we have described in our study.
(6 KB TXT).

Data S3 Source Code for Simulation Study 1 This file contains the R source code that we used to perform the first simulation study in our paper.
(31 KB TXT).

Data S4 Source Code for Simulation Study 2 This file contains the R source code that we used to perform the second simulation study in our paper.
(39 KB TXT).

Dataset S1 Breast Cancer Expression Data: van't Veer et al. (2002) Study The gene expression data for the breast cancer study of van't Veer et al. (2002). We include only the expression levels of 4,751 genes identified in the study whose expression varied
(2.9 MB CSV).

Dataset S2 Breast Cancer Gene Names: van't Veer et al. (2002) Study The names of each of the 4,751 genes in the study of van't Veer et al. (2002).
(74 KB CSV).

Dataset S3 Breast Cancer Survival Data: van't Veer et al. (2002) Study The clinical data for the study of van't Veer et al. (2002). The first column represents the time until metastasis (or the time until the patient left the study); the second column is 1 if the tumor metastasized and 0 if it did not.
(1 KB CSV).

Dataset S4 Breast Cancer Expression Data: van de Vijver et al. (2002) Study The gene expression data for the 70 genes in the breast cancer study of van de Vijver et al. (2002).
(141 KB CSV).

Dataset S5 Breast Cancer Gene Names: van de Vijver et al. (2002) Study The names of the 70 genes in the study of van de Vijver et al. (2002).
(1 KB CSV).

Dataset S6 Repeated Breast Cancer Samples A single column that is 1 if the patient was included in the earlier study (that of van't Veer et al. [2002]), and 0 if the patient was not included in the earlier study.
(1 KB CSV).

Dataset S7 Breast Cancer Survival Data: van de Vijver et al. (2002) Study The clinical data for the study of van de Vijver et al. (2002). The format is the same as the format of the earlier file of clinical data.
(5 KB CSV).

Dataset S8 DLBCL Expression Data The gene expression data for the DLBCL study of Rosenwald et al. (2002).
(24.38 MB CSV).

Dataset S9 DLBCL Survival Data The clinical data for the study of Rosenwald et al. (2002). The format is the same as the format of the clinical data above.
(2 KB CSV).

Dataset S10 Lung Cancer Gene Expression Data This is the gene expression data for the lung cancer dataset of Beer et al. (2002).
(5.39 MB TXT).

Dataset S11 Lung Cancer Survival Data This is the clinical data for the lung cancer dataset of Beer et al. (2002). The format is the same as in the clinical data above.
(1 KB TXT).

Dataset S12 AML Gene Expression Data This is the gene expression data for the AML dataset of Bullinger et al. (2004).
(9.96 MB TXT).

Dataset S13 AML Survival Data This is the clinical data for the AML dataset of Bullinger et al. (2004). It has the same format as the clinical data above.
(1 KB TXT).

Figure S1 Results of Using PLS-Derived Cox Scores in the Supervised Clustering Procedure (8.26 MB TIFF).

Figure S2 Plot of Survival Versus the Least Squares Estimate of β˜ for the DLBCL Data (8.33 MB TIFF).

Figure S3 Plot of Survival Versus the Least Squares Estimate of γ˜ for the DLBCL Data (8.42 MB TIFF).

Protocol S1 Additional Models and Methods (28 KB TEX).

## Abbreviations

DLBCL: diffuse large B-cell lymphoma
LDA: linear discriminant analysis
PLS: partial least squares
SVM: support vector machine

## References

[pbio-0020108-Alizadeh1] Alizadeh AA, Eisen MB, Davis RE, Ma C, Lossos IS (2000). Distinct types of diffuse large B-cell lymphoma identified by gene expression profiling. Nature.

[pbio-0020108-Beer1] Beer DG, Kardia SL, Huang CC, Giordano TJ, Levin AM (2002). Gene-expression profiles predict survival of patients with lung adenocarcinoma. Nat Med.

[pbio-0020108-Ben-Dor1] Ben-Dor A, Friedman N, Yakhini Z (2001). Class discovery in gene expression data. Proceedings of the fifth annual international conference on computational biology.

[pbio-0020108-Bhattacharjee1] Bhattacharjee A, Richards WG, Staunton J, Li C, Monti S (2001). Classification of human lung carcinomas by mRNA expression profiling reveals distinct adenocarcinoma subclasses. Proc Natl Acad Sci U S A.

[pbio-0020108-Bullinger1] Bullinger L, Döhner K, Bair E, Fröhling S, Schlenk R (2004). Gene expression profiling identifies new subclasses and improves outcome prediction in adult myeloid leukemia. N Engl J Med.

[pbio-0020108-Chu1] Chu G, Narasimhan B, Tibshirani R, Tusher V (2002). Significance analysis of microarrays (PAM) software. http://www-stat.stanford.edu/tibs/SAM/.

[pbio-0020108-Coiffier1] Coiffier B (2001). Diffuse large cell lymphoma. Curr Opin Oncol.

[pbio-0020108-Cox1] Cox DR, Oakes D (1984). Analysis of survival data.

[pbio-0020108-Eisen1] Eisen MB, Spellman PT, Brown PO, Botstein D (1998). Cluster analysis and display of genome-wide expression patterns. Proc Natl Acad Sci U S A.

[pbio-0020108-Golub1] Golub T, Slonim DK, Tamayo P, Huard C, Gaasenbeek M (1999). Molecular classification of cancer: Class discovery and class prediction by gene expression monitoring. Science.

[pbio-0020108-Gordon1] Gordon AD (1999). Classification.

[pbio-0020108-Hastie1] Hastie T, Tibshirani R, Botstein D, Brown P (2001a). Supervised harvesting of expression trees. Genome Biol.

[pbio-0020108-Hastie2] Hastie T, Tibshirani R, Friedman J (2001b). The elements of statistical learning: Data mining, inference and prediction.

[pbio-0020108-Hedenfalk1] Hedenfalk I, Duggan D, Chen Y, Radmacher M, Bittner M (2001). Gene-expression profiles in hereditary breast cancer. N Engl J Med.

[pbio-0020108-Horn1] Horn RA, Johnson CR (1985). Matrix analysis.

[pbio-0020108-Khan1] Khan J, Wei JS, Ringnér M, Saal LH, Ladanyi M (2001). Classification and diagnostic prediction of cancers using gene expression profiling and artificial neural networks. Nat Med.

[pbio-0020108-Lapointe1] Lapointe J, Li C, van de Rijn M, Huggins JP, Bair E (2004). Gene expression profiling identifies clinically relevant subtypes of prostate cancer. Proc Natl Acad Sci U S A.

[pbio-0020108-Lehmann1] Lehmann E, Casella G (1998). Theory of point estimation.

[pbio-0020108-Li1] Li H, Luan Y (2003). Kernel Cox regression models for linking gene expression profiles to censored survival data. Pacific symposium on biocomputing. http://www.smi.stanford.edu/projects/helix/psb03/li.pdf.

[pbio-0020108-McLachlan1] McLachlan GJ (1992). Discriminant analysis and statistical pattern recognition.

[pbio-0020108-Nguyen1] Nguyen DV, Rocke DM (2002a). Multi-class cancer classification via partial least squares with gene expression profiles. Bioinformatics.

[pbio-0020108-Nguyen2] Nguyen DV, Rocke DM (2002b). Tumor classification by partial least squares using microarray gene expression data. Bioinformatics.

[pbio-0020108-NHLCP1] [NHLCP] Non-Hodgkin's Lymphoma Classification Project (1997). A clinical evaluation of the international lymphoma study group classification of non-hodgkin's lymphoma. Blood.

[pbio-0020108-Nutt1] Nutt CL, Mani DR, Betensky RA, Tamayo P, Cairncross JG (2003). Gene expression-based classification of malignant gliomas correlates better with survival than histological classification. Cancer Res.

[pbio-0020108-Ramaswamy1] Ramaswamy S, Tamayo P, Rifkin R, Mukherjee S, Yeang CH (2001). Multiclass cancer diagnosis using tumor gene expression signatures. Proc Natl Acad Sci U S A.

[pbio-0020108-Rosenwald1] Rosenwald A, Wright G, Chan WC, Connors JM, Campo E (2002). The use of molecular profiling to predict survival after chemotherapy for diffuse large B-cell lymphoma. N Engl J Med.

[pbio-0020108-Shipp1] Shipp MA, Ross KN, Tamayo P, Weng AP, Kutok JL (2002). Diffuse large B-cell lymphoma outcome prediction by gene-expression profiling and supervised machine learning. Nat Med.

[pbio-0020108-Sorlie1] Sorlie T, Perou C, Tibshirani R, Aas T, Geisler S (2001). Gene expression patterns of breast carcinomas distinguish tumor subclasses with clinical implications. Proc Natl Acad Sci U S A.

[pbio-0020108-Therneau1] Therneau TM, Grambsch PM (2000). Modelling survival data: Extending the cox model.

[pbio-0020108-Tibshirani1] Tibshirani R, Hastie T, Narasimhan B, Chu G (2002). Diagnosis of multiple cancer types by shrunken centroids of gene expression. Proc Natl Acad Sci U S A.

[pbio-0020108-Tibshirani2] Tibshirani R, Hastie T, Narasimhan B, Chu G (2003). Prediction analysis for microarrays (PAM) software. http://www-stat.stanford.edu/tibs/PAM/index.html.

[pbio-0020108-Tusher1] Tusher VG, Tibshirani R, Chu G (2001). Significance analysis of microarrays applied to the ionizing radiation response. Proc Natl Acad Sci U S A.

[pbio-0020108-vannde1] van de Vijver MJ, He YD, van 't Veer LJ, Dai H, Hart AA (2002). A gene-expression signature as a predictor of survival in breast cancer. N Engl J Med.

[pbio-0020108-vant1] van't Veer LJ, Dai H, van de Vijver MJ, He YD, Hart AAM (2002). Gene expression profiling predicts clinical outcome of breast cancer. Nature.

[pbio-0020108-von1] von Heydebreck A, Huber W, Poustka A, Vingron M (2001). Identifying splits with clear separation: A new class discovery method for gene expression data. Bioinformatics.

[pbio-0020108-Vose1] Vose JM (1998). Current approaches to the management of non-hodgkin's lymphoma. Semin Oncol.

