# Supplementary figures and images for: Semi-Supervised Methods to Predict Patient Survival from Gene Expression Data
Source: PLoS Biol. 2004 Apr 13;2(4):e108. doi: 10.1371/journal.pbio.0020108 (PMC387275; doi:10.1371/journal.pbio.0020108)

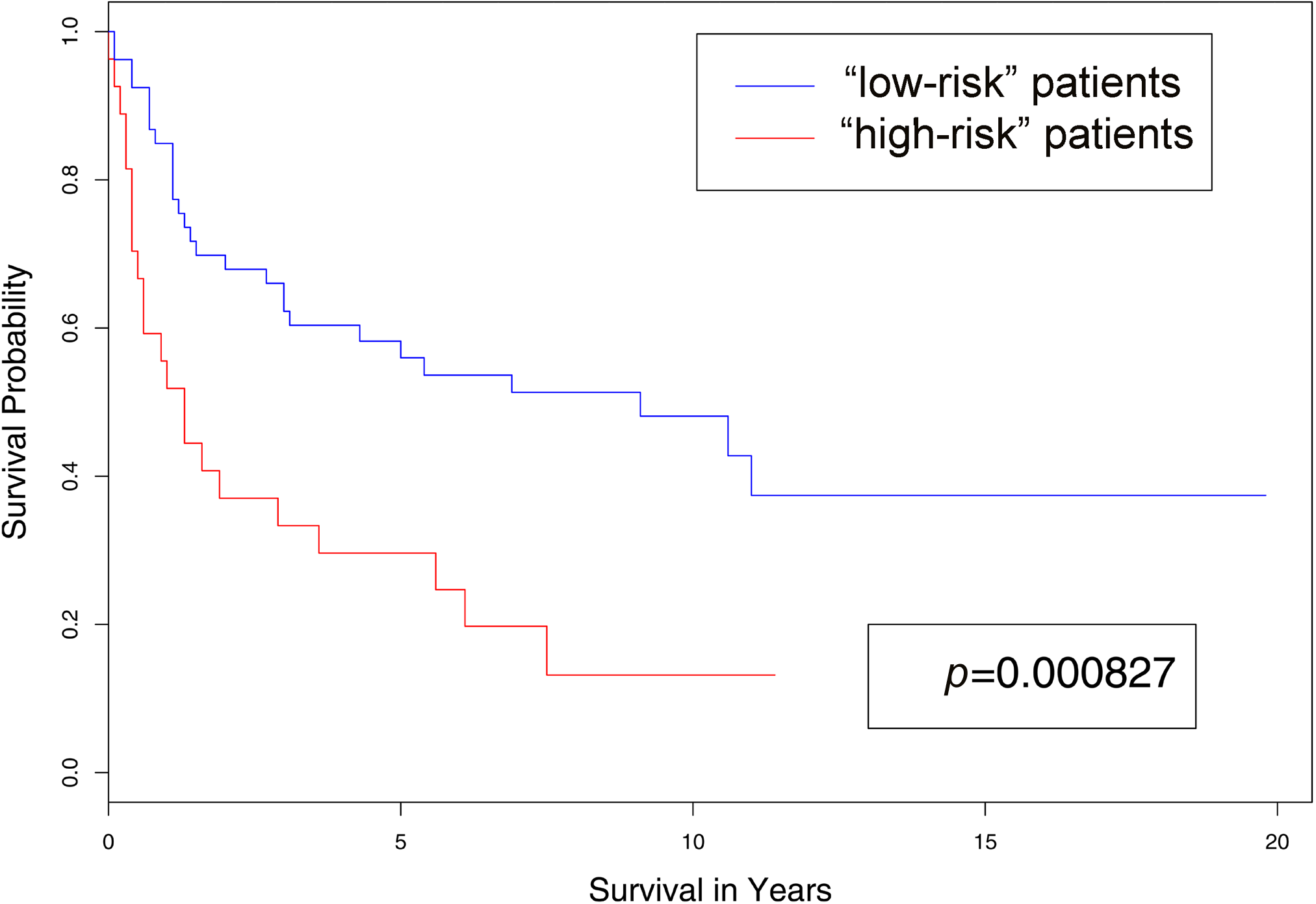

Supplement: Figure S1 — (8.26 MB TIFF). [file pbio.0020108.sg001.tif]

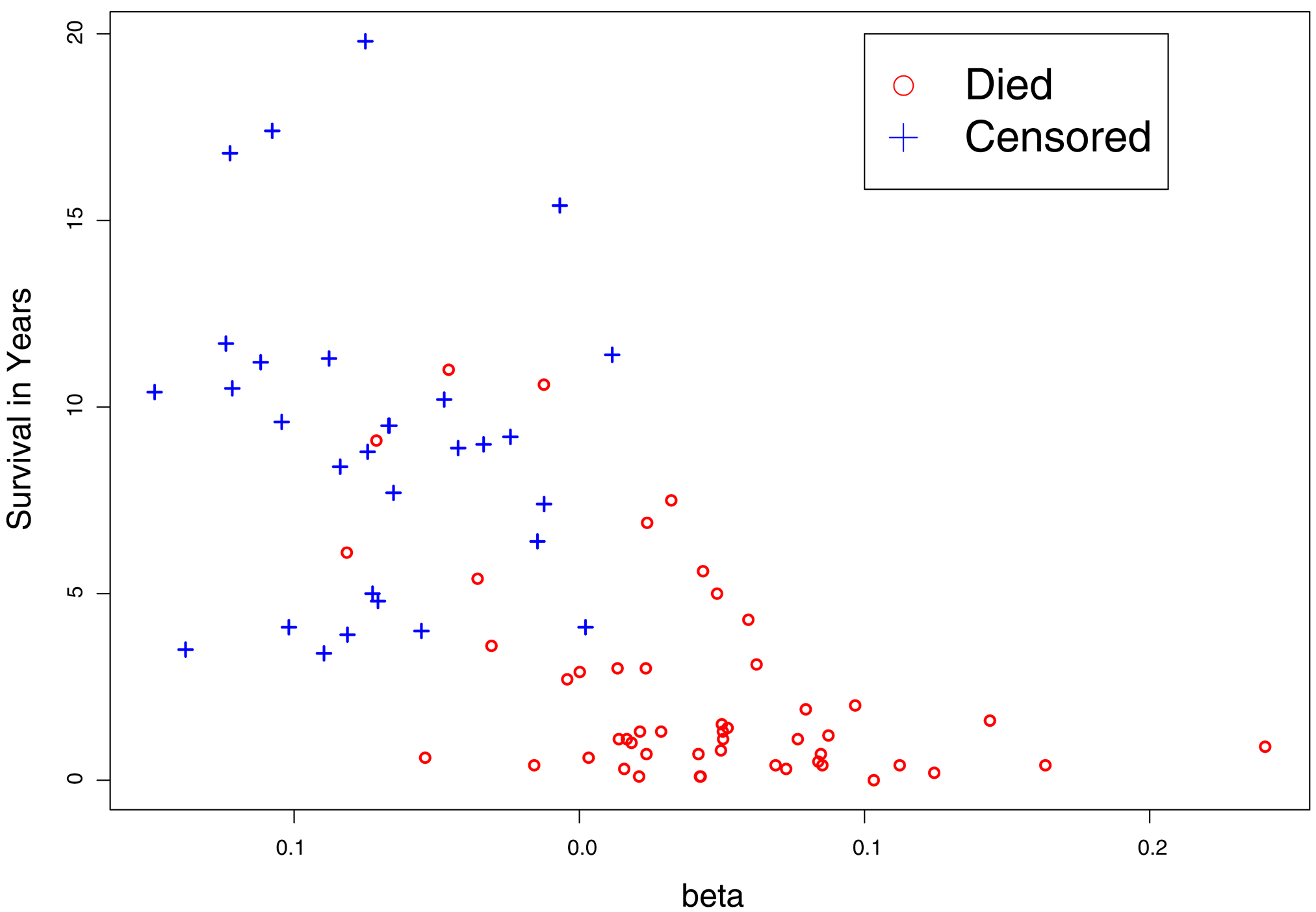

Supplement: Figure S2 — (8.33 MB TIFF). [file pbio.0020108.sg002.tif]

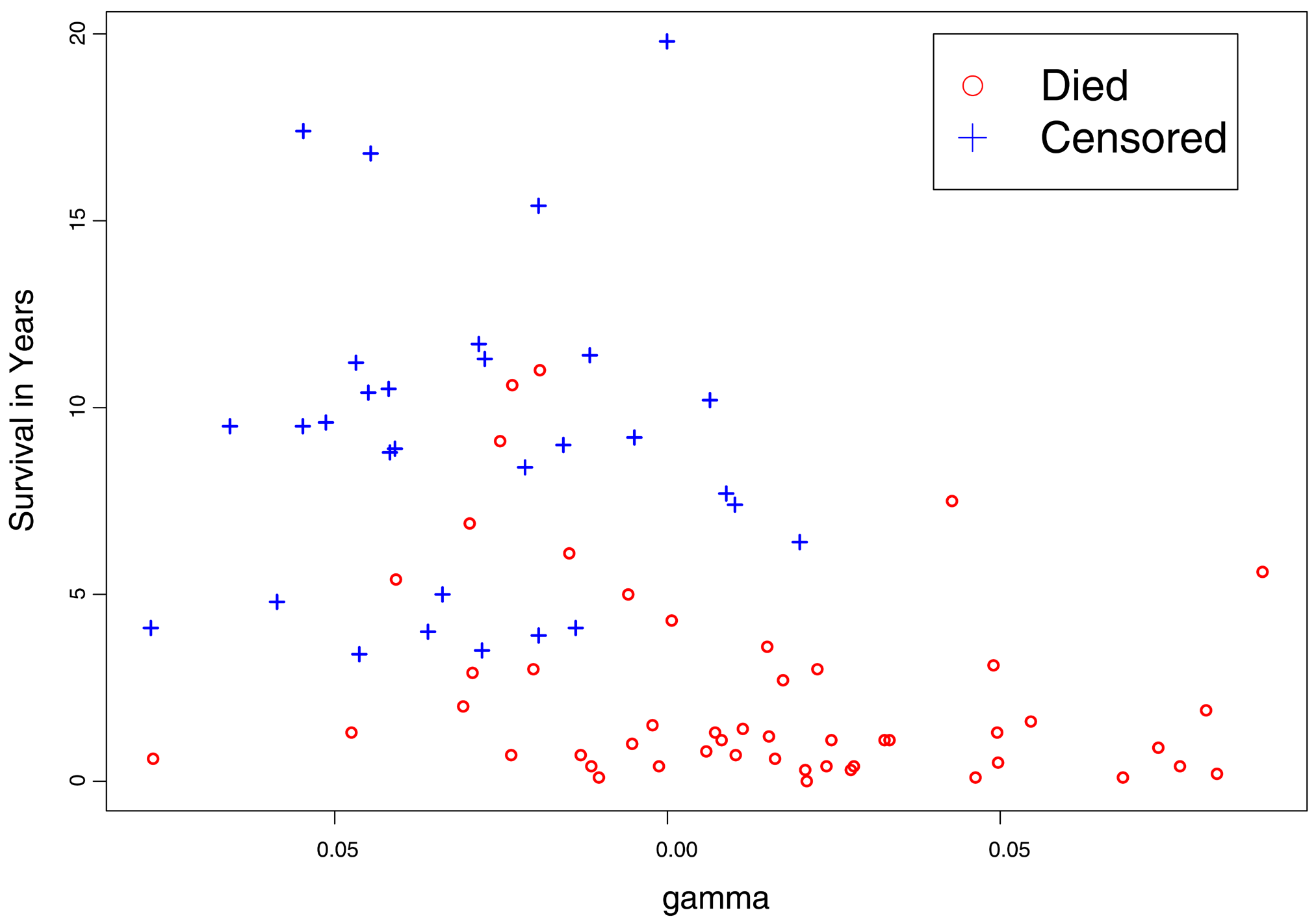

Supplement: Figure S3 — (8.42 MB TIFF). [file pbio.0020108.sg003.tif]
